# Emphysematous gastritis: The silent storm within the stomach wall

**DOI:** 10.1177/03000605251389384

**Published:** 2025-10-31

**Authors:** Chander Shekher Aggarwal, Simran Dahiya, Smriti Kochhar, Kanishk Aggarwal, Luke Edgecombe, Toluwanimi Jeje, Rohit Jain

**Affiliations:** 1 Department of Internal Medicine, White River Health, USA; 2 Department of Medicine, Maulana Azad Medical College, India; 3 Department of Medicine, Penn State Milton S. Hershey Medical Center, USA; 4 Dayanand Medical College and Hospital, India; 5 Department of Surgery, White River Health, USA; 6 Department of Medicine, 432137 Avalon University School of Medicine, 432137 Curaçao

**Keywords:** Emphysematous gastritis, gas-forming microorganisms, esophagogastroduodenoscopy, gastric perforation, peritonitis, gastrointestinal bleeding

## Abstract

Emphysematous gastritis is a rare, life-threatening condition characterized by inflammation of the stomach lining and gas within the gastric wall, with a mortality rate ranging from 55% to 61%. Emphysematous gastritis is caused by gas-producing bacteria and fungi that can infect both gastric and bowel walls. Risk factors include diabetes, corticosteroid use, prolonged nonsteroidal anti-inflammatory drug use, neoplasia, recent abdominal surgery, and other conditions that compromise immune function. Emphysematous gastritis can present with nonspecific symptoms such as abdominal pain, nausea, vomiting, diarrhea, and fever; however, severe manifestations such as hematemesis and sepsis may also occur. Symptoms alone are insufficient for establishing a definitive diagnosis; computed tomography is the most effective imaging modality to identify characteristic findings. Management typically involves conservative treatment with bowel rest, hydration, and intravenous antibiotics. Surgical intervention may be required in cases of clinical deterioration; however, its role remains controversial. Recent studies have questioned the necessity of surgery, emphasizing early medical management to improve outcomes. This narrative review aims to provide an overview of emphysematous gastritis, focusing on its pathophysiology, clinical features, diagnostic methods, and treatment strategies. It highlights the significance of timely intervention to improve survival and reduce the high mortality associated with this rare condition.

## Introduction

Emphysematous gastritis (EG) is a rare and potentially life-threatening condition characterized by inflammation of the gastric lining and gas within the gastric wall. It represents a severe form of gastric pneumatosis, which includes a range of conditions, from benign conditions such as gastric emphysema (GE) to EG.^1^ A concise comparison of EG and GE is presented in Table 1,^1,2^ which highlights their distinguishing features. EG has a reported mortality rate of 55%–61% and was first described by Fraenkel in 1889.^3,4^ Although gas is normally present within the lumen of bowel loops, its presence in the parenchyma of solid organs or the walls of hollow viscera is considered pathological.^5^

**Table 1.** Comparison of the key features of emphysematous gastritis and gastric emphysema.^1,2^

Feature	Emphysematous gastritis	Gastric emphysema
Cause	Infectious (gas-producing bacteria)	Noninfectious (ischemia, trauma, vomiting)
Onset	Acute, severe	Often mild, incidental
Symptoms	Pain, vomiting, fever, hemodynamic instability	Pain, vomiting, stable vitals
Mortality	>50%	<30%
CT findings	Streaky/mottled air, wall thickening	Round air bubbles
Endoscopy	Variable findings, mucosal necrosis, ulceration, and purulent drainage	Redness, erosion, coarse mucosa, ulceration, clear demarcation between normal and abnormal mucosa
Management	Conservative treatment and/or surgery if deteriorating	Mostly conservative treatment

EG is most commonly caused by gas-producing gram-positive and gram-negative bacteria and occasionally by fungi. Patients often present with nonspecific symptoms such as abdominal pain, nausea, vomiting, diarrhea, and fever, with some progressing to more severe manifestations such as hematemesis and sepsis. Such nonspecific presentation makes diagnosis challenging, particularly in advanced stages, emphasizing the need for early detection and treatment.^6^ Symptoms alone are insufficient for diagnosis; computed tomography (CT) is the most effective diagnostic tool for EG.^7^ Treatment options may include conservative management or surgical resection of the stomach, depending on the patient’s clinical status.^8^ This narrative review summarizes the pathophysiology, clinical features, diagnosis, and treatment of EG.

## Methods

This narrative review summarizes the current evidence on the pathophysiology, clinical features, diagnostic approaches, and management of EG following the Scale for the Assessment of Narrative Review Articles (SANRA) guidelines.^9^ A comprehensive literature search was conducted across PubMed, Scopus, DynaMed, and Google Scholar for English-language studies published up to August 2025 using the search terms “emphysematous gastritis,” “gastric pneumatosis,” “gas-forming infections of the stomach,” and “management of emphysematous gastritis.” References of retrieved articles were also screened. Eligible studies included case reports, case series, retrospective analyses, review articles, and systematic reviews that discuss the etiology, pathogenesis, clinical presentation, diagnosis, and treatment of EG. Studies focused solely on GE, pediatric populations, or unrelated gastric disorders were excluded unless they provided comparative data. Two authors independently extracted data on patient demographics, risk factors, causative organisms, imaging findings, endoscopic features, therapeutic interventions, and outcomes. Discrepancies were resolved through discussion, and data were summarized narratively and presented in tables, where appropriate. The primary outcomes of interest were mortality rates, clinical recovery, and complications associated with EG. Secondary outcomes included the diagnostic accuracy of imaging modalities and effectiveness of conservative versus surgical management approaches.

### Clinical presentation

EG causes severe systemic impairment by releasing endotoxins and inflammatory mediators. Common symptoms include vomiting, diarrhea, abdominal pain, ileus, leukocytosis, fever, and hemodynamic instability. According to a systematic review of 121 patients by Elnaggar et al., the most common symptoms were abdominal pain (90%) and vomiting, nausea, and diarrhea (80%).^10^ In immunocompromised individuals, such as those with diabetes and renal or hepatic failure, the condition may manifest subacutely with only fever or milder symptoms.^11^ In rare cases, it can present with sudden epigastric pain, hematemesis, or melena.^3^ Complications include gastric perforation, septic shock, gastrointestinal bleeding, abscess formation, and disseminated infection.^12^ Prognosis depends on early recognition and timely intervention, often involving antibiotic use, supportive care, and, in some cases, surgical debridement.

### Pathophysiology

The pathophysiology of GE, which can progress to EG, involves a complex interplay of predisposing factors, including mucosal injury, microbial invasion, ischemic changes, and immune system compromise.^13^ The stomach’s mucosal barrier, comprising a thick layer of mucus, tight epithelial junctions, and a robust blood supply, serves as a critical defense mechanism against microbial invasion.^14^ However, any disruption to this barrier, due to ulceration, trauma, instrumentation, or use of medications such as nonsteroidal anti-inflammatory drugs (NSAIDs) and corticosteroids, predisposes the stomach wall to microbial infiltration. In addition, alcohol consumption and smoking can impair the gastric blood supply, weaken mucosal defenses, and increase susceptibility to bacterial invasion.^6^ Once the mucosal barrier is compromised, gas-forming microorganisms, particularly *Escherichia coli, Klebsiella pneumoniae, Staphylococcus aureus, Clostridium perfringens, Enterococcus* species, and *Streptococcus* species can infiltrate the stomach wall. In immunocompromised patients, fungal pathogens such as *Candida* and *Aspergillus* species may also be involved.^15^

These microorganisms produce enzymes such as urease and protease, which generate gases such as nitrogen, hydrogen, and carbon dioxide that accumulate in the gastric mucosa.^15^ Increased intraluminal pressure, as observed in conditions such as pyloric obstruction and severe vomiting, can facilitate deeper penetration of these organisms into the tissue. Loss of epithelial integrity and poor perfusion from septic shock, cardiac arrest, or severe dehydration further facilitate this invasion. Ischemia exacerbates the process by impairing immune responses and creating a hypoxic condition that favors the proliferation of anaerobic bacteria, such as *Clostridium* species.^16,17^

**Figure 1.**
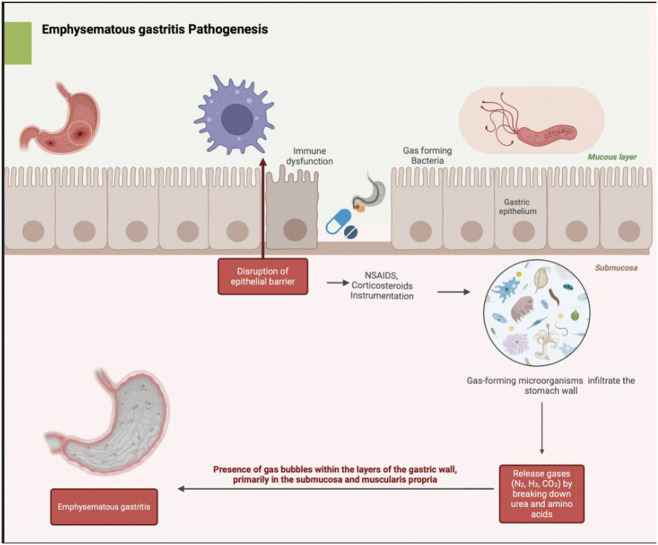
Pathogenesis of emphysematous gastritis showing mucosal injury, microbial invasion, and gas production within the gastric wall, leading to intramural gas accumulation in the submucosa and muscularis propria.

Chronic illnesses such as diabetes and kidney disease as well as immunosuppressive therapy weaken host defenses through impaired neutrophil and phagocyte function, increasing the risk of EG.^18^ Typical features include gas bubbles in the submucosa and muscularis propria, accompanied with necrosis caused by bacterial toxins and ischemia.^7^ This disrupts the gastric architecture and function. Neutrophil and macrophage infiltration causes fluid buildup, thereby weakening the wall and increasing the risk of perforation.^19^ This has been demonstrated visually in Figure 1.

## Discussion

### Trends in EG management

The mechanisms underlying EG highlight the complex interplay between microbial invasion, tissue ischemia, and systemic inflammation. Building on this foundation, the following section explores the infectious agents and predisposing conditions responsible for the development of EG as well as the evolving approaches to diagnosis and treatment.

### Infectious etiologies and predisposing conditions

A retrospective analysis by Watson et al. reviewed 59 adult cases of EG documented before 2013 and identified *Clostridium* species, *Streptococcus* species, *Escherichia coli, Klebsiella*, and *Pseudomonas* as the most common causative organisms.^20^ Similar trends were noted by Ono et al. in their analysis of 40 cases published between 2019 and 2022, with a rare case caused by *Bacteroides uniformis*.^18^ Fungal pathogens such as *Candida* and Mucorales have also been reported as causative agents, with their effects attributed to their angio-invasive properties.^13^^,21^ *Sarcina* ventriculi, also known as *Clostridium ventriculi*, is a rare causative organism of EG found on gastric biopsy in two cases.^22^ Al Rasheed and Senseng reported *Sarcina* in patients with delayed gastric emptying, emphasizing its association with severe complications.^22^ Elnaggar et al., in their systematic review of 121 cases, observed that *Sarcina ventriculi* was the most frequently reported gas-producing microorganism associated with gastric wall infections.^10^

These organisms have been identified through gastric aspirates or tissue biopsy culture. In some cases, no organism was isolated. Watson et al. found that no causative organism was isolated in 25 of the 59 cases (42.4%) reviewed.^20^ Although the precise pathogenesis of EG remains unclear, it is believed to involve mucosal injury and systemic illness. Contributing factors include alcohol abuse, ingestion of corrosive substances, abdominal surgery, and NSAID use.^21^

In a recent case report, Fatima et al. described a young male patient without any comorbid conditions who developed EG following a binge-drinking episode.^23^ A comprehensive literature review conducted by Matsushima et al. included 39 EG cases reported up to 2012; they found that diabetes mellitus was the most common predisposing factor, with a positive medical history in 10 patients. A history of malignancy was noted in five patients, while renal failure and organ transplant were implicated in seven and three cases, respectively.^15^ Although EG is commonly observed in individuals with underlying predisposing conditions, a few rare cases have been reported in otherwise healthy adults. Yu et al. reported a case of EG in a young, healthy man, possibly caused by infection secondary to the consumption of contaminated deer meat while hunting.^24^ In another case involving a young, healthy man with no prior comorbidities, EG developed after the consumption of pickles a day before symptom onset. It was hypothesized that pickle juice, an acid-containing food item, contributes to histopathological changes in the gastric mucosa, creating a conducive environment for the gas-forming bacteria to invade and cause infection.^25^

In immunocompromised patients, such as those with diabetes, renal failure, or cirrhosis, the presentation of EG may be more severe and rapid. These patients are at higher risk of systemic sepsis and multiorgan failure due to their impaired immune status. For instance, diabetic patients may present with altered mental status and severe leukocytosis, as seen in the case of an 82-year-old diabetic man. Similarly, patients with renal failure, such as those on hemodialysis, may exhibit more pronounced systemic toxicity and a higher risk of mortality.^26,27^

Patients with cirrhosis may present with additional complications, such as hepatic encephalopathy and worsening hepatic function, as seen in the case of a patient who developed EG status post partial hepatectomy. The presence of portal venous gas in these patients can further complicate the clinical picture.^28^

Collectively, these cases highlight several key trends in the epidemiology and clinical spectrum of EG. Bacterial pathogens, particularly *Clostridium, Streptococcus, Sarcina ventriculi*, and *E. coli*, remain the most commonly identified causative organisms; however, fungal infections are increasingly being recognized in immunocompromised patients.^10,13,20,21^ Most cases occur in individuals with underlying conditions such as diabetes, renal failure, malignancy, and recent surgery; however, a subset of reports have documented EG in previously healthy adults, often following mucosal insult from alcohol or acidic food intake.^26,27^ The variability in causative organisms, absence of identifiable pathogens in some cases, and the broad range of predisposing factors underscore the heterogeneous nature of EG and the importance of maintaining a high index of suspicion in both high-risk and atypical patient populations.

### Diagnostic approach and management

EG presents with nonspecific symptoms such as fever, abdominal pain, nausea, vomiting, and diarrhea, making radiological imaging essential for differentiating it from its more benign predecessor, GE. Both conditions are characterized by the presence of gas in the gastric wall; however, in GE, this typically appears as thin, linear intramural air collections, while in EG, it presents as cystic gas pockets and is associated with a considerably worse prognosis.^12^

EG is very rare; therefore, there are no established guidelines for the diagnosis and management of EG patients. However, most case reports published to date emphasize prompt diagnosis and aggressive treatment.^29^ Although plain radiographs and ultrasound can detect air in the gastric mucosa, they have low sensitivity and specificity. CT is the definitive imaging modality for diagnosing EG because it can confirm the intramural location of the gas and the extent of mucosal involvement.^5^ CT characteristically demonstrates the presence of cystic air pockets trapped within the gastric wall, hypertrophic mucosal folds, and free air within the peritoneal cavity and portal vein.^30^ The presence of portal venous gas is a poor prognostic indicator, associated with 75% mortality, and approximately all patients require surgical management.^31^ According to a comprehensive review, CT was performed for 31 of the 39 cases reported between 1982 and 2009, of which 41% showed radiographic findings of intramural gas in the stomach.^15^

Esophagogastroduodenoscopy (EGD) plays an essential role in assessing the extent of mucosal damage and obtaining tissue biopsies for microbiological analysis in treatment-resistant cases.^7^ Matsushima et al. found that EGD was performed in 48.7% of suspected EG cases, and >50% of these cases exhibited erosion of the gastric mucosa and necrosis of the stomach wall.^15^ In a recent systematic review, endoscopy revealed gastric necrosis in 40% of the cases and inflammation in 30%, characterized by mucosal erythema and edema. Ulcerations were observed in 25% of the cases, while perforation occurred in 10%, often linked to ischemic damage. Normal or nonspecific findings were reported in 10% of the cases, usually due to incomplete evaluations.^10^ EGD is primarily indicated when signs of severe sepsis are present or when microbiologic confirmation is required.^7^

Management depends on the severity of the presentation. Patients with normal-appearing mucosa benefit from conservative measures such as bowel rest, intravenous (IV) fluid administration, and close hemodynamic monitoring. Patients should be started on broad-spectrum antibiotics covering gram-negative and anaerobic bacteria; antifungal agents may be added to the treatment regimen if fungal etiology is suspected.^32^ Surgery is indicated in patients with evidence of perforation, stricture, or clinical deterioration despite medical therapy.^15,33^

Although surgery may be lifesaving, it is associated with postoperative complications, including bleeding, worsening infection, anastomotic leak, bowel obstruction, ileus, ischemia, fistula and stricture formation, adhesions, stenosis, and wound dehiscence, all contributing to increased morbidity and mortality.^34,35^ There are no set protocols for the duration of antibiotic therapy in these patients. Yusef et al., based on their study on a patient with EG, reported the effectiveness of a 14-day course of antimicrobial treatment.^36^

After assessing cases of EG published from 2019 to 2022, Ono et al. found that surgical treatment was performed for 13 of the 40 patients, while the remaining majority was managed conservatively.^18^ Matsushima et al. and Watson et al. similarly noted that the use of surgical intervention has declined over time, with laparotomies performed in 62.5% of the cases before 2000 versus only 22.2% after 2000.^15,20^ According to a systematic review by Elnaggar et al., involving 121 cases of EG, management primarily involved medical therapy, which was used in 75% of cases, with antibiotics such as vancomycin, piperacillin-tazobactam, and metronidazole, along with supportive care. Nasogastric decompression was used in 11% of the patients. Surgical intervention was required in 25% of the cases; the most common surgical intervention was gastrectomy (32%), followed by laparotomy (21%) and splenectomy (4%), reconfirming the trend toward conservative management. Overall, the recovery rate was 70%, with a mortality rate of 15%.^10^

Long-term sequelae of EG remain poorly understood. Jenkins et al. hypothesized that EG sequelae involve connective tissue hyperplasia accompanied with residual fibrosis. Reportedly, 25% of surviving patients developed gastric contractures. The clinical implications of this outcome remain unclear; potential concerns include malabsorptive conditions and chronic pain.^12^

A clear contrast emerges between conservative and surgical management. Conservative therapy, consisting of bowel rest, broad-spectrum antibiotics, proton pump inhibitors, and supportive care, has shown favorable outcomes in hemodynamically stable patients without evidence of perforation or necrosis, as reported by Nasser et al. and Sánchez-Hernández et al.^4,11^ In contrast, surgical intervention is typically reserved for cases with extensive gastric necrosis, perforation, or uncontrolled sepsis. These patients often have a poorer prognosis, with high mortality rates reported in studies by Huang et al. and Costa et al.^30,34^ The shift from early aggressive surgical management to an initial conservative approach reflects improvements in diagnostic imaging, early recognition, and antibiotic therapy.

In summary, EG is an extremely rare and potentially life-threatening condition, with only a few cases documented in the literature. The cases analyzed in this review are summarized in Table 2. The scarcity of data and published studies presented several challenges in the preparation of this review. Nevertheless, the aim was to consolidate the available information into a single, comprehensive resource for researchers, clinicians, and students. We hope this review not only serves as a consolidated reference but also contributes toward shaping future understanding and management strategies for EG.

**Table 2.** Comparison of key features, pathogens identified, management, and outcomes from published cases.

First author (year)	Patient presentation	Pathogen(s) identified	Management	Outcome
Shipman PJ (2001)^16^	A 27-year-old woman presented with epigastric pain that had been aggravating for 2 days. Examination revealed hypotension, fever, and epigastric tenderness. Abdomen CT demonstrated gas within the peripheral portal veins of the liver and irregular submucosal gas within a thickened gastric wall.	*Enterococcus* species	Managed conservatively with broad-spectrum antibiotics	The patient was discharged after a protracted recovery.
Jung JH (2007)^13^	A 43-year-old man presented with diffuse abdominal pain, indigestion, and poor oral intake for 4 days. Abdominal CT showed gastric wall thickening with dirty air bubbles and a small pneumoperitoneum, consistent with emphysematous gastritis (EG) and perforation.	*Klebsiella pneumoniae, Staphylococcus aureus,* and *Pseudomonas aeruginosa.* *Klebsiella pneumoniae, Staphylococcus aureus, Pseudomonas aeruginosa,* and mucormycosis	Surgically managed with total gastrectomy and intravenous (IV) antibiotics, followed by IV antifungal postoperatively. Reoperation was performed due to wound dehiscence, anastomotic leakage, and necrosis of the transverse colon and liver. Roux-en-Y esophagojejunostomy with cecocolostomy and segmental resection of the large intestine were performed.	Despite aggressive management, the patient developed multiple organ necrosis, postoperative abscess, and respiratory failure. He died on hospital day 21.
Loi TH (2007)^28^	A 45-year-old Chinese woman presented with rising alpha-fetoprotein levels and a palpable liver 6 cm below the costal margin. Abdomen CT showed a large heterogeneous mass in the right lobe of the liver, and she underwent a right hepatectomy for hepatocellular carcinoma. On postoperative day 8, she developed jaundice with murky abdominal drain output, leukocytosis, and worsening liver function. Ultrasound revealed partial thrombosis of the right portal vein, and CT showed an edematous stomach wall with air pockets within the thickened wall.	*Pseudomonas* and *Acinetobacter* species	Treated with IV antibiotics	The patient improved and was discharged home.
Huang CT (2009)^30^	An 83-year-old woman presented with a 4-day history of anorexia, malaise, and vomiting. Twenty days prior, she had sustained a left pubic ramus fracture treated conservatively with narcotic analgesics. Initially diagnosed with narcotic-induced ileus, her symptoms worsened, progressing to coffee-ground emesis and melena. CT showed extensive pneumatosis in the stomach and small intestine, diffusely dilated small bowel loops, and gas in the superior mesenteric vein extending into the portal vein.	*Escherichia coli*	Treated with IV antibiotics and emergent total gastrectomy with segmental small bowel resection.	Despite intervention, the patient’s hemodynamic status deteriorated, and she died on hospital day 7.
Rankin I (2013)^31^	Case 1: An 81-year-old man presented with severe right upper quadrant and epigastric pain radiating to the back, which had worsened over 12 h. He was febrile, tachycardic, and tachypneic. CT revealed extensive hepatic portal venous gas with branching air-filled structures extending to the periphery of the liver, gas within several upper abdominal mesenteric veins, and gas in the posterior gastric wall, consistent with gastric ischemia.	None	Managed conservatively with IV antibiotics and IV pantoprazole.	The patient was discharged with outpatient follow-up.
	Case 2: A 74-year-old Caucasian woman presented with a 3-day history of generalized abdominal pain, vomiting, diarrhea, and bleeding per rectum. Abdomen CT with contrast showed widespread bowel ischemia with gas in the portal vein as well as extensive gas in the liver, stomach wall, draining veins, gallbladder, common bile duct, and most of the small bowel walls.	*Citrobacter koseri*	Managed conservatively with IV antibiotics and pantoprazole. No surgical intervention was performed due to the extent of ischemia.	The patient died 36 h after admission.
Ratuapli SK (2013)^21^	A 73-year-old man with a history of medically refractory gastric ulcers status post antrectomy with gastrojejunostomy (Billroth II) and truncal vagotomy in 1985 presented for evaluation of iron deficiency anemia. He was asymptomatic with no gastrointestinal complaints. Endoscopy revealed gastric erythema, a food bezoar, and features of chronic gastritis.	*Sarcina ventriculi*	Managed conservatively with IV antibiotics and sucralfate along with IV and oral iron supplementation.	The patient’s symptoms improved, and follow-up endoscopy after 3 months showed resolution of gastric erythema.
Yusef D (2014)^36^	A 16-year-old male patient presented with hematemesis, melena, and abdominal pain. He had a gastrostomy in place and a recent right orchidectomy for infarcted testis, for which he was administered oral antibiotics for 5 days. Abdomen/pelvis CT showed thickening of the distal esophagus and stomach with diffuse intramural gas. Esophagogastroduodenoscopy (EGD) revealed severe esophageal candidiasis and inflammation around the gastroesophageal junction without evidence of perforation.	None	Managed conservatively with IV fluids, omeprazole, packed red cell transfusion, intravenous antibiotics, and antifungal.	The patient survived and tolerated full enteral feeding by day 7.
Nasser H (2019)^4^	Case 1: A 78-year-old woman presented with a 1-week history of diffuse abdominal pain, nausea, vomiting, and diarrhea. Abdomen CT revealed gastric distension with gastric pneumatosis,	None	Managed conservatively with nasogastric tube decompression, IV fluids, bowel rest, proton pump inhibitor (PPI), and broad-spectrum antibiotics.	The patient improved clinically and was discharged home on PPI therapy with re-initiation of warfarin.
	Case 2: An 87-year-old woman presented with a 4-day history of diffuse abdominal pain and non-bilious, non-bloody emesis. Abdomen CT without contrast showed gastric pneumatosis with portal venous gas throughout the liver.	None	Managed conservatively with bowel rest, pump inhibitor (PPI) therapy, and IV antibiotics.	She completed 7 days of antibiotics, tolerated oral intake, and was discharged home on PPI therapy.
	Case 3: A 78-year-old woman from a long-term acute care facility presented with a 2-day history of mild diffuse abdominal pain, nausea, and vomiting. Abdomen CT revealed gastric pneumatosis with free intraperitoneal air.	None	Managed conservatively with nasogastric tube decompression, bowel rest, IV fluids, PPI therapy, and IV antibiotics.	The patient’s symptoms improved, and she was discharged back to her long-term acute care facility after a 1-week antibiotic course. Two months later, she was admitted to another hospital and died from multiorgan failure of unclear etiology.
Weaver A (2019)^32^	A 73-year-old man presented with abdominal pain, weakness, nausea, several episodes of non-bloody vomiting, and frequent loose stools. Abdomen CT with contrast revealed air in the portal venous system and air within the gastric fundus extending into the perigastric vessels, without free air in the abdomen.	None	Conservative management with IV antibiotics and transition to oral antibiotics after discharge.	The patient was discharged from the hospital on day 5 and remained symptom-free at the 2-week follow-up.
Costa AC (2020)^34^	A 54-year-old woman presented with epigastric pain, hematemesis, and melena. Abdominal CT showed marked gastric distension with mottled gas in the gastric wall, consistent with EG, without portal venous gas.	*Escherichia coli*	Underwent lifesaving total gastrectomy with esophago-jejunal anastomosis.	The patient died the following week due to anastomotic dehiscence.
Riaz S (2020)^3^	A 96-year-old man presented with acute-onset epigastric discomfort and coffee-ground emesis. Abdomen CT showed gastric wall thickening with gas, followed by EG, which showed gastric wall necrosis. Gastric biopsy showed EG.	None	Managed conservatively with nasogastric tube gastric decompression, IV pantoprazole, broad-spectrum IV antibiotics, and IV fluids.	The patient was discharged from the hospital after 10 days
Sánchez-Hernández C (2022)^11^	A 78-year-old man presented with a pertrochanteric fracture of the left hip; her hospital course was complicated by abdominal distention with decreased peristalsis in addition to arterial hypotension. Abdominal CT showed evidence of gastric dilation and multiple air bubbles in the wall.	None	Managed conservatively with a broad-spectrum antibiotic, fluid therapy, and parenteral nutrition.	He recovered fully and was discharged.
Ono R (2022)^18^	An 80-year-old woman was admitted with an acute myocardial infarction and underwent stent implantation for mid-left anterior descending artery stenosis. Two weeks after admission, while on tube feeding, she developed vomiting followed by epigastric pain. Examination revealed abdominal distension and epigastric tenderness without peritoneal signs. Abdomen CT revealed air in the gastric wall with hepatic portal venous gas, without involvement of other intestinal tracts or arterial flow impairment.	*Bacteroides uniformis*	Managed conservatively with IV antibiotics and nutrition.	Follow-up CT after 2 days showed resolution of portal venous gas, and the patient remained stable after completing 2 weeks of antibiotic therapy.
Jenkins JK (2023)^12^	A 77-year-old man presented on day 10 after his final cycle of R-CHOP chemotherapy with fever, rigors, and vomiting. Abdomen/pelvis CT revealed significant gastric intramural gas extending into the portal venous system and liver, consistent with EG.	*Pseudomonas aeruginosa*	Managed conservatively with complete bowel rest, parenteral nutrition, broad-spectrum antibiotics, PPI therapy, and the addition of IV metronidazole for anaerobic coverage. Surgical intervention was deemed futile.	The patient showed slow but steady improvement. He was extubated on day 10, and by day 13, a repeat CT demonstrated full resolution of gastric and portal venous air. He was transferred to the ward on day 14 and later to a rehabilitation facility, showing significant recovery by day 40.
Qasim A (2023)^7^	Case 1: A 35-year-old man presented with a 2-day history of diffuse abdominal pain, non-bilious vomiting with blood specks, diarrhea, fever with chills, dry cough, and nasal congestion. Respiratory viral panel was positive for rhino/enterovirus. Abdomen/pelvis CT demonstrated circumferential gastric wall thickening with multiple small intramural air collections and mild perigastric fat stranding.	*Rhino/enterovirus*	Managed conservatively with nasogastric tube decompression, IV fluids, IV pantoprazole, and IV antibiotics along with IV metoclopramide.	The patient tolerated oral feeding. He was discharged home after 5 days.
	Case 2: An 81-year-old man from a nursing home was admitted with a 3-day history of abdominal pain, 1-day history of nausea and vomiting, and episodes of coffee-ground emesis. A prior endoscopy had shown grade C esophagitis and chronic mild gastritis. Abdomen/pelvis CT revealed a distended stomach with questionable pneumatosis in the fundus, portal venous gas, and ascending colon wall thickening, along with severe fecal impaction. Findings were consistent with EG.	None	Managed conservatively with nasogastric tube decompression, IV fluids, IV pantoprazole, and IV antibiotics. Aggressive bowel management with enemas, lactulose, senna, docusate, and manual disimpaction was performed.	The patient was discharged back to the nursing home.
Bhosale SJ (2024)^8^	A 15-year-old male patient presented on day 13 of treatment with abdominal pain, respiratory distress, and multiple loose stools. CT scan showed gastric wall thickening with intramural gas in the fundal region, consistent with EG. Endoscopy was not performed due to hemodynamic instability.	*Staphylococcus aureus, Streptococcus pneumoniae, Pseudomonas aeruginosa,* and *Klebsiella pneumoniae*	Managed conservatively with IV antibiotics, antifungal, and supportive care. Surgical intervention was deferred.	Despite aggressive management, the patient’s hemodynamic status deteriorated, and he died on day 3 after ICU admission.
Elnaggar M (2025)^10^	This systematic review included 121 patients from 116 published case reports and case series spanning the years 1947–2024. Patient ages ranged from 4 months to 96 years, with an average age of 55 years. There was a slight male predominance, with 66 male and 55 female patients diagnosed with EG. Endoscopy revealed gastric necrosis in 40% of cases and inflammation in 30%, marked by mucosal erythema and edema. Ulcerations were observed in 25%, while perforation occurred in 10%, often linked to ischemic damage. Normal or nonspecific findings were reported in 10% patients, mostly due to incomplete evaluations.	*Sarcina ventriculi* was the most frequently reported gas-producing microorganism associated with gastric wall infections.	Management primarily involved medical therapy, used in 75% of cases, with antibiotics such as vancomycin, piperacillin-tazobactam, and metronidazole, along with supportive care. Nasogastric decompression was used in 11% of the patients. Surgical intervention was required in 25% of the cases; gastrectomy (32%) was performed most frequently, followed by laparotomy (21%) and splenectomy (4%).	Overall, the recovery rate was 70%, with a mortality rate of 15%.
Nahar S (2025)^37^	A 55-year-old man, presented with severe epigastric pain, nausea, and approximately 100 episodes of non-bloody vomiting in a single day. He also reported loose stools, chills, and diaphoresis; however, he denied having fever, hematemesis, or melena. Abdomen/pelvis CT showed moderate distension of the fluid-filled stomach and gas within the stomach wall, consistent with gastric pneumatosis. A hiatal hernia and fatty liver were also noted; however, the pancreas and other abdominal organs appeared normal. EGD was not performed during hospitalization.	None	Managed conservatively without surgery. He was treated with IV piperacillin/tazobactam for suspected infectious gastritis and IV pantoprazole for acid suppression. IV fluids and electrolyte replacement (potassium and sodium) were given to correct dehydration and imbalances. Ondansetron was used for nausea relief. As his symptoms improved, he was transitioned to a clear liquid diet, followed by a regular diet without issues.	The patient was discharged in stable condition on oral pantoprazole, augmentin, potassium supplements, and PRN antiemetics.
Naidoo D (2025)^38^	Case 1: An 82-year-old man with multiple comorbidities (CHF, COPD, CKD, hemiparesis, and erosive gastritis) presented with nausea, vomiting, diarrhea, abdominal pain, and shortness of breath. He was hypotensive and bradycardic in the field. On examination, he had abdominal distension and tenderness. Abdomen CT (noncontrast) showed gastric wall thickening with pneumatosis and portal venous gas, consistent with EG. No perforation or abscess was seen. EGD was not performed.	None	The patient was treated conservatively with nasogastric decompression, IV fluids, IV antibiotics (cephalosporin, metronidazole, fluconazole), and pain management. No surgical intervention was required.	The patient’s symptoms gradually improved. The leukocyte count normalized, and he tolerated oral feeding. He was discharged home after 4 days of conservative treatment with oral antibiotics.
	Case 2: An 81-year-old man with gastric adenocarcinoma and recent blood transfusion presented with diffuse abdominal pain, bilateral lower extremity nonpruritic rash, and fever. Examination showed abdominal tenderness, black guaiac-positive stool, and a purpuric rash from the feet to knees. Abdomen CT with contrast revealed gastric pneumatosis, extensive portal and mesenteric venous gas, and pneumoperitoneum, indicating EG. There was no clear site of perforation, but findings were consistent with severe gastric ischemia or necrosis. No endoscopy (EGD) was performed due to rapid deterioration.	None	Initial management included IV fluids, IV antibiotics (cefepime, metronidazole), and pantoprazole. Despite aggressive care, the patient rapidly decompensated within hours, developing worsening hypotension and lactic acidosis.	Due to poor prognosis and metastatic cancer, no surgery was offered. The patient was transitioned to comfort care and died within 6 hours of arrival at the ED.
Ghadban E (2025)^39^	An 87-year-old Middle Eastern woman presented with diffuse abdominal pain and severe postprandial vomiting for 1 week. Physical examination showed abdominal distension with epigastric tenderness, but no guarding. Abdomen CT (noncontrast) revealed significant gastric distension, gastric and esophageal stasis, portal venous gas, gas within the stomach wall (pneumatosis), and features suggestive of superior mesenteric artery (SMA) syndrome. EGD showed severely distended gastric cavity, multiple fundal ulcers, and fragile erythematous mucosa, with inability to pass beyond the pylorus—likely due to extrinsic compression. Findings suggested possible gastric ischemia.	None	The patient was initially managed conservatively with nasogastric decompression, NPO status, IV fluids, and broad-spectrum antibiotics. Due to persistent vomiting and confirmed duodenal obstruction on barium swallow, exploratory surgery was performed. Intraoperatively, there was no evidence of necrosis; however, SMA syndrome was confirmed. A gastrojejunostomy was performed using an omega loop and GIA stapler to bypass the obstruction.	The patient was discharged home in stable condition and was doing well at the 1-week follow-up, continuing her routine hemodialysis without complications. The surgical intervention proved effective after initial conservative measures failed to fully resolve symptoms.

CT: computed tomography; ICU: intensive care unit; PRN: pro re nata; R-CHOP: rituximab–cyclophosphamide–hydroxydaunorubicin–oncovin–prednisone; CHF: chronic heart failure; CKD: chronic kidney disease; COPD: chronic obstructive pulmonary disease; NPO: nil per OS; IV: intravenous; ED: emergency department; GIA: gastrointestinal anastomosis.

## Conclusion

EG is a rare, life-threatening condition caused by gas-forming organisms that infect the gastric wall, often in the presence of risk factors such as diabetes, immunosuppression, and mucosal injury. Although surgery is historically the primary treatment option, recent trends favor conservative management, including bowel rest, IV fluid administration, and broad-spectrum antibiotics, especially in the absence of ischemia. However, there is no standardized diagnostic pathway or clear criteria for selecting surgical versus nonsurgical approaches. The rarity and variability of EG continue to hinder the development of evidence-based protocols. To address these gaps, multicenter research and consensus guidelines are needed to facilitate early diagnosis, guide treatment decisions, and reduce the morbidity and mortality associated with EG.

## Data Availability

Not applicable to this article because no datasets were generated or analyzed during the current study.
